# Shift in soil organic carbon and nitrogen pools in different reclaimed lands following intensive coastal reclamation on the coasts of eastern China

**DOI:** 10.1038/s41598-019-42048-6

**Published:** 2019-04-11

**Authors:** Wen Yang, Lu Xia, Zhihong Zhu, Lifen Jiang, Xiaoli Cheng, Shuqing An

**Affiliations:** 1 College of Life Sciences, Shaanxi Normal University, Xi’an, 710119 P. R. China; 2 School of Life Science and Institute of Wetland Ecology, Nanjing University, Nanjing, 210023 P. R. China; 3 Center for Ecosystem Science and Society (Ecoss), Department of Biological Sciences, Northern Arizona University, Flagstaff, AZ 86011 USA; 4 Key Laboratory of Aquatic Botany and Watershed Ecology, Wuhan Botanical Garden, Chinese Academy of Sciences, Wuhan, 430074 P. R. China

## Abstract

The impacts of coastal reclamation on carbon (C) and nitrogen (N) sinks of coastal wetlands remain unclearly understood. This study was conducted to investigate the alterations of soil organic C and N (SOC and SON) pools following conversion of *Phragmites australis* salt marsh into fishpond, wheat and rapeseed fields and town construction land through reclamation along Jiangsu coast in eastern China. Coastal reclamation significantly increased stocks of soil total, labile and recalcitrant organic C and N (SLOC, SLON, SROC, and SRON), and concentrations of water-soluble organic C (WSOC), microbial biomass C and N (SMBC and SMBN), cumulative CO_2_-C mineralization (MINC) following conversion of *P. australis* salt marsh into fishpond, wheat and rapeseed fields. However, coastal reclamation reduced SOC, SLOC, SROC, SRON, WSOC, SMBC, SMBN, and MINC following conversion of *P. australis* salt marsh into town construction land. Our results suggest that coastal reclamation affects C and N sinks of coastal wetlands by changing SOC and SON pools size, stability and dynamics changes following conversion of *P. australis* salt marsh into other land use types. This finding were primarily attributed to alterations in quantity and quality of exogenous materials returning the soil, and soil physiochemical properties as affected by coastal reclamation.

## Introduction

Coastal reclamation has become a prevailing and rapid approach to alleviate the constraints of land resources and the increasing need for living space for human beings^1^. In the past, many coastal countries, including the Netherlands^2^ and United States^3^ have conducted intensive coastal reclamation for urbanization, and agriculture and mariculture^4^. In China, coastal reclamation resulted in the loss of 13,380 km^2^ of coastal wetlands and 73% of mangroves during 1950–2008, and area of loss up to half of natural coastal wetlands^4^. In accordance with new coastal development strategies of China, approximately 5780 km^2^ of coastal wetlands will be converted to other land use types by 2020^1^. However, coastal reclamation has been reported to bring serious implications for marine and coastal ecosystems, such as loss of coastal habitats and biodiversity^5^, leading to the deterioration of marine environments^6^, declining ecosystem service function^4^, and accelerating landscape fragmentation^4^.

Coastal wetlands are considered as the crucial part of ‘blue carbon (C)’ sinks due to high primary productivity with low rates of soil organic C (SOC) decomposition^7^, and also play a significant role in global C cycle with positive feedback to global climate change^8,9^. Coastal reclamation can greatly modify hydrodynamics, morphology, and sediment transportation of coasts^10^, and further alter soil physicochemical properties^4^. Generally, the alterations of land use patterns, plant community composition and productivity, as well as soil management practices are important ecological drivers for SOC and soil organic nitrogen (SON) sequestration^11,12^. Whereas, in salt-affected reclaimed soils, SOC and SON sequestration have not only been affected by general factors but also by specific soil physicochemical properties, especially salinity and alkalinity^12,13^. Salinity and alkalinity have been demonstrated to negatively affect SOC and SON accumulation through inhibiting plant growth, thus lessening plant materials entering the soil^12^, and decreasing soil organic matter (SOM) decomposition by restricting soil microbial activity^14^. Although some studies have reported that the impacts of coastal reclamation on SOC and SON pools^12,15–18^, there are no unanimous conclusion. For instance, Zhang *et al*.^18^ reported that soil C and N sequestration greatly increased following conversion of coastal wetlands into farmlands through rapid desalination and dealkalization. Contrarily, Han *et al*.^19^ exhibited that the conversion of coastal wetlands to farmlands and other land use types would decrease soil C sequestration and accelerate C emission through changing anaerobic environment in the wetlands. These inconsistent results were probably induced by diverse variations in land use types, reclamation history, intensity and duration, and soil management practices^4^. Thus, identifying the influences of coastal reclamation to SOC and SON sequestration as well as associated driving factors are urgently needed.

Soil organic matter (SOM) is comprised of various functional soil fractions with distinct degrees of stability and turnover times^20^. Chemical fractionation techniques can be used to separate organic C and N of bulk soil into different functional organic C and N pools, i.e., soil labile organic C and N (SLOC and SLON, respectively), and recalcitrant organic C and N (SROC and SRON, respectively)^21,22^. SLOC and SLON pools are the available nutrient stores with the small size and extremely high biological activity^23^, which are more sensitive to environmental changes than the total SOC and SON pools and thus have been considered as an early indicator of variations in SOC and SON pools induced by different land use practices^12^. SLOC and SLON pools consist of different fractions, such as water-soluble organic C (WSOC), soil microbial biomass C and N (SMBC and SMBN, respectively), and cumulative CO_2_-C mineralization (MINC), and these soil fractions can be used to indicate the changes of soil C and N dynamics^24^. SROC and SRON pools have larger sizes, and are recognized as the most stable SOC and SON owing to their longer residence times and lower turnover rates^22^, which dominate long-term C and N storage^23,25^. Hence, a comprehensive understanding of the variations of SLOC (WSOC, MBC, MBN, and MINC), SLON, SROC, as well as SRON, and their driving mechanism following coastal reclamation have important implications for evaluating SOC and SON pools size, stability and dynamics changes in coastal wetlands.

In eastern China, Jiangsu province has the largest area of coastal wetlands^26^, and is considered as a hotspot region of coastal reclamation^27^. The total area of reclamation in Jiangsu province was approximately 1769.82 km^2^ from 1979 to 2014^27^. The natural coastal wetlands in Jiangsu are characterized by bare flat, *Spartina alterniflora*, *Suaeda salsa*, and *Phragmites australis* salt marshes^24^. Currently, the vast majority of coastal wetlands have been reclaimed and converted into aquaculture ponds (e.g., fishpond), farmlands (e.g., wheat and rapeseed fields), as well as town construction lands which are the primary land use patterns of coastal wetlands along Jiangsu’s coasts^26^, especially in Dafeng and Sheyang counties of the middle Jiangsu coast^28^. Previous studies have documented that the responses of SOC and SON to coastal reclamation^15–18^. However, those studies focused mostly on the changes of total SOC and SON^15–18^, while the functional organic C and N pools (i.e., SLOC, SLON, SROC, and SRON) received little attention. The impacts of coastal reclamation on SLOC, SLON, SROC, and SRON pools following conversion of coastal wetlands into aquaculture ponds, farmlands, and town construction lands are still unknown. We hypothesize that coastal reclamation can alter SLOC, SLON, SROC, and SRON pools through changing soil physiochemical properties as well as exogenous materials entering the soil following conversion of coastal wetlands into aquaculture ponds, farmlands, and town construction lands. To test this, we examined concentrations and stocks of SOC, SON, SLOC, SROC, SLON, SRON, recalcitrant indices for C (RIC) and N (RIN), and the concentrations of WSOC, SMBC, SMBN, MINC, as well as soil physiochemical properties (i.e., soil moisture, bulk density, pH and salinity) in coastal reclaimed fishpond, wheat field, rapeseed field, and town construction lands in comparison to adjacent *P. australis* salt marsh. The objectives of this study were to: (1) evaluate whether the responses of SOC, SON, and various functional organic C and N pools to coastal reclamation would differ among different land use types; (2) which land use type had the greatest SOC and SON accumulation, and the most stable SOC and SON pools, respectively; and (3) identify which important factor could drive the changes in SOC and SON pools size, stability and dynamics following coastal reclamation.

## Results

### Soil and plant properties

**Figure 1:**
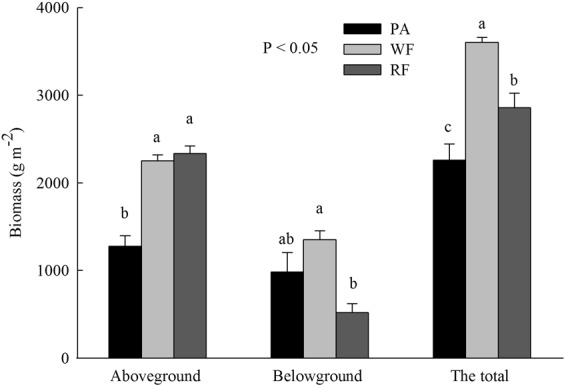
The aboveground, belowground (0–30 cm soil depth), and the total biomass of *P. australis* salt marsh, wheat field and the rape field. Different letters over the bars indicate statistically significant differences at α = 0.05 level among *P. australis* salt marsh, wheat field and the rape field. PA: *P. australis* salt marsh; WF: Wheat field; RF: Rapeseed field.

Soil/sediment moisture was highest in fishpond, intermediate in wheat field, rapeseed field and *P. australis* salt marsh, and lowest in town construction land (Table 1). Rapeseed field revealed higher soil bulk density compared with other land use types (Table 1). The maximum and minimum soil/sediment pH was found in *P. australis* salt marsh and fishpond, respectively (Table 1). Soil/sediment salinity was highest in *P. australis* salt marsh followed by fishpond, rapeseed field, wheat field, and town construction land (Table 1). The aboveground biomass in wheat and rapeseed fields was significantly higher than that in *P. australis* salt marsh (Fig. 1). The highest and lowest belowground biomass were showed in wheat field and rapeseed field, respectively (Fig. 1). The total biomass in wheat field was considerably higher than that in rapeseed field and *P. australis* salt marsh (Fig. 1).

**Table 1:** Soil physiochemical properties (mean ± SE, n = 12) in different land use types in the Jiangsu coast of eastern China.

Land use types	Moisture (%)	BD (g cm^−3^)	pH	Salinity (%)
*Phragmites australis* salt marsh	25.55 ± 0.37^c^	1.31 ± 0.03^b^	8.83 ± 0.06^a^	0.53 ± 0.03^a^
Fishpond	49.87 ± 1.11^a^	1.07 ± 0.01^c^	7.44 ± 0.05^e^	0.31 ± 0.04^b^
Wheat field	31.80 ± 1.03^b^	1.24 ± 0.05^b^	7.90 ± 0.06^d^	0.07 ± 0.02^d^
Rapeseed field	30.12 ± 1.20^b^	1.51 ± 0.08^a^	8.17 ± 0.02^c^	0.18 ± 0.01^c^
Town construction land	22.33 ± 0.07^d^	1.21 ± 0.02^bc^	8.60 ± 0.01^b^	0.03 ± 0.01^d^
Source of variation				
Coastal reclamation	^***^	^***^	^***^	^***^

Different superscript lower case letters indicate statistically significant differences at the α = 0.05 level among land use types. ^***^P < 0.001. BD: bulk density.

### C and N concentrations and stocks of soil pool

**Figure 2:**
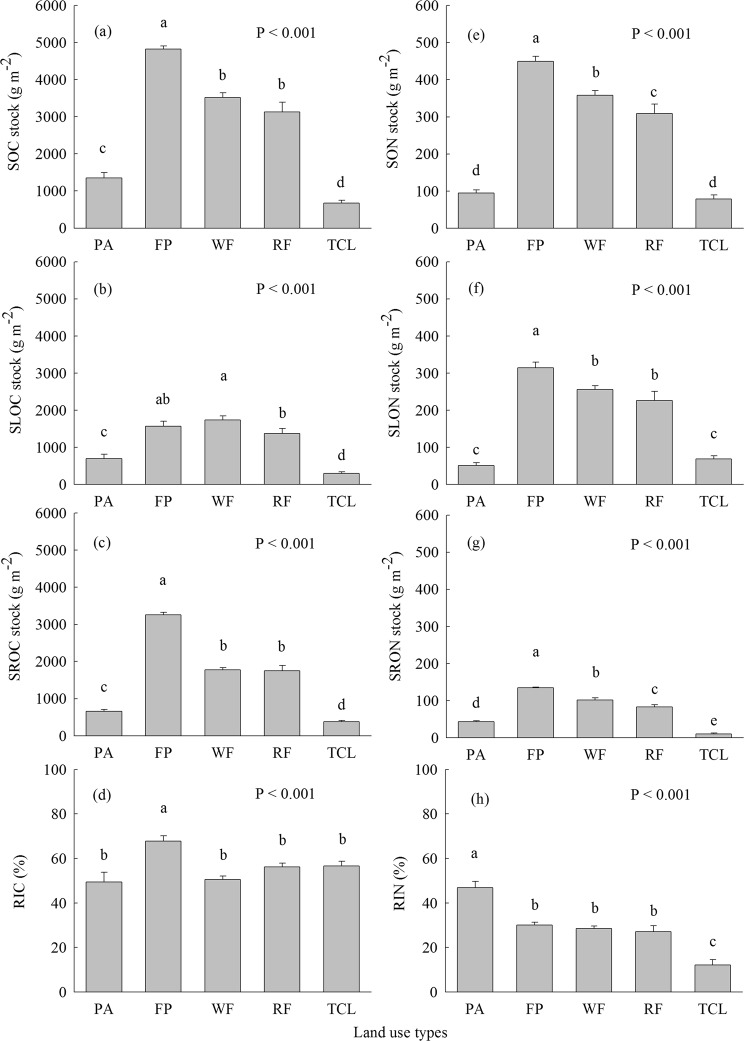
(**a**) Soil organic carbon (SOC), (**b**) soil labile organic carbon (SLOC), (**c**) soil recalcitrant organic carbon (SROC), (**d**) recalcitrance index for carbon (RIC), (**e**) Soil organic nitrogen (SON), (**f**) soil labile organic nitrogen (SLON), (**g**) soil recalcitrant organic nitrogen (SRON), and (**h**) recalcitrance index for nitrogen (RIN) of different land use types. Different letters over the bars indicate statistically significant differences at α = 0.05 level among land use types. FP: Fishpond; TCL: Town construction land. See Fig. 1 for abbreviations.

The concentrations and stocks of SOC, SROC, SON, SLON, as well as SRON were highest in fishpond followed by wheat field and rapeseed field compared with *P. australis* salt marsh and town construction land (Table 2; Fig. 2). The concentrations and stocks of SLOC were highest in wheat field and fishpond, and lowest in town construction land (Table 2; Fig. 2). The concentrations and stocks of SOC, SLOC, SROC, and SRON in *P. australis* salt marsh were significantly higher than those in town construction land (Table 2; Fig. 2).

**Table 2:** The concentrations of soil total, labile, and recalcitrant organic C and N (mean ± SE, n = 12) in different land use types in the Jiangsu coast of eastern China.

Land use types	Soil organic carbon pool			Soil organic nitrogen pool		
	SOC (g kg^−1^)	SLOC (g kg^−1^)	SROC (g kg^−1^)	SON (g kg^−1^)	SLON (g kg^−1^)	SRON (g kg^−1^)
*Phragmites australis* salt marsh	3.43 ± 0.33^d^	1.77 ± 0.28^c^	1.66 ± 0.09^d^	0.239 ± 0.018^d^	0.128 ± 0.016^d^	0.111 ± 0.004^d^
Fishpond	15.07 ± 0.36^a^	4.89 ± 0.46^a^	10.18 ± 0.21^a^	1.403 ± 0.047^a^	0.983 ± 0.050^a^	0.420 ± 0.005^a^
Wheat field	9.50 ± 0.24^b^	4.70 ± 0.24^a^	4.80 ± 0.12^b^	0.967 ± 0.021^b^	0.692 ± 0.024^b^	0.275 ± 0.010^b^
Rapeseed field	6.90 ± 0.29^c^	3.02 ± 0.17^b^	3.88 ± 0.19^c^	0.682 ± 0.022^c^	0.497 ± 0.028^c^	0.185 ± 0.017^c^
Town construction land	1.86 ± 0.23^e^	0.82 ± 0.13^d^	1.04 ± 0.10^e^	0.219 ± 0.029^d^	0.191 ± 0.023^d^	0.028 ± 0.008^e^
Source of variation						
Coastal reclamation	^***^	^***^	^***^	^***^	^***^	^***^

Different superscript lower case letters indicate statistically significant differences at the α = 0.05 level among land use types. ^***^P < 0.001. SOC: soil organic carbon; SLOC: soil labile organic carbon; SROC: soil recalcitrant organic carbon; SON: soil organic nitrogen; SLON: soil labile organic nitrogen; SRON: soil recalcitrant organic nitrogen.

The stocks of SOC, SLOC, and SROC ranged from 673–4822 g m^−2^, 296–1739 g m^−2^, as well as 377–3258 g m^−2^ among land use types, respectively (Fig. 2). The stocks of SOC, SLOC, and SROC in fishpond, wheat field and rapeseed field, after coastal reclamation, increased by 1.31- to 2.56-fold, 0.97- to 1.49-fold, and 1.67- to 3.96-fold in comparison with *P. australis* salt marsh, respectively (Fig. 2). Whereas, the stocks of SOC, SLOC, and SROC in town construction land decreased by 0.50-fold, 0.58-fold, and 0.43-fold relative to *P. australis* salt marsh (Fig. 2). The stocks of SON, SLON, and SRON ranged from 79–449 g m^−2^, 51–315 g m^−2^, as well as 10–135 g m^−2^ among land use types, respectively (Fig. 2). The stocks of SON, SLON, and SRON in fishpond, wheat field and rapeseed field increased by 2.26- to 3.74-fold, 3.43- to 5.17-fold, and 0.88- to 2.06-fold in comparison to *P. australis* salt marsh, respectively (Fig. 2). Soil/sediment RIC ratio in fishpond was significantly higher than that in other land use types (Fig. 2d). The highest and lowest RIN ratios were found in *P. australis* salt marsh and town construction land, respectively (Fig. 2h).

### Dynamics of SOC and SON

**Figure 3:**
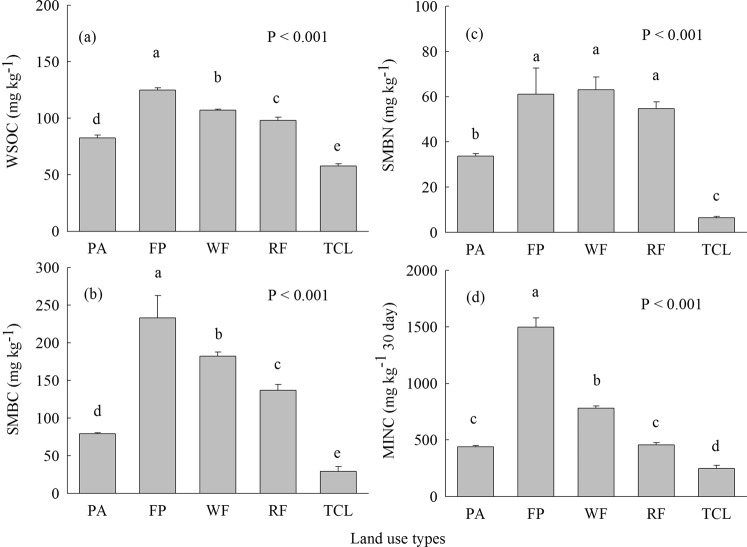
(**a**) Soil water-soluble organic carbon (WSOC), (**b**) soil microbial biomass carbon (SMBC), (**c**) soil microbial biomass nitrogen (SMBN), and (**d**) cumulative CO_2_-C mineralization (MINC) of different land use types. Different letters over the bars indicate statistically significant differences at α = 0.05 level among land use types. See Figs. 1 and 2 for abbreviations.

The concentrations of WSOC, SMBC, and SMBN in fishpond, wheat field and rapeseed field were considerably higher than those in *P. australis* salt marsh (Fig. 3a–c). The highest WSOC, SMBC, and MINC concentrations were observed in fishpond among land use types (Fig. 3a,b,d). The concentrations of WSOC, SMBC, SMBN, as well as MINC were lowest in town construction land among land use types (Fig. 3).

### Linking SOC and SON pools to soil and plant properties

The pearson’s correlation analysis revealed that the variations in SOC, SLOC, SROC, SON, SLON, SRON, WSOC, SMBC, SMBN, and MINC concentrations were highly related to soil moisture, while were negatively correlated with soil pH (Table 3). Linear regression analysis indicated that the concentrations of SOC, SLOC, SROC, SON, SLON, SRON, WSOC, SMBC, and SMBN among *P. australis* salt marsh, wheat and rapeseed fields showed a significant positive correlation with aboveground as well as total plant biomass (Table 4).

**Table 3:** Correlation analysis of soil physiochemical properties, and soil C and N fractions across different land use types.

	Moisture	BD	pH	Salinity	SOC	SLOC	SROC	SON	SLON	SRON	WSOC	SMBC	SMBN
SOC	0.949^**^	−0.402	−0.954^**^	−0.069	1								
SLOC	0.783^**^	−0.249	−0.890^**^	−0.024	0.918^**^	1							
SROC	0.974^**^	−0.455^**^	−0.927^**^	−0.088	0.979^**^	0.818^**^	1						
SON	0.924^**^	−0.384	−0.976^**^	−0.048	0.990^**^	0.927^**^	0.960^**^	1					
SLON	0.901^**^	−0.365	−0.981^**^	−0.117	0.974^**^	0.921^**^	0.939^**^	0.994^**^	1				
SRON	0.939^**^	−0.412	−0.921^**^	−0.115	0.985^**^	0.900^**^	0.967^**^	0.968^**^	0.937^**^	1			
WSOC	0.860^**^	−0.191	−0.847^**^	−0.176	0.935^**^	0.894^**^	0.897^**^	0.921^**^	0.888^**^	0.958^**^	1		
SMBC	0.863^**^	−0.300	−0.890^**^	−0.109	0.937^**^	0.939^**^	0.877^**^	0.934^**^	0.917^**^	0.933^**^	0.912^**^	1	
SMBN	0.626^**^	−0.049	−0.690^**^	−0.146	0.752^**^	0.852^**^	0.654^**^	0.754^**^	0.734^**^	0.768^**^	0.830^**^	0.896^**^	1
MINC	0.967^**^	−0.556^*^	−0.878^**^	−0.208	0.955^**^	0.828^**^	0.960^**^	0.929^**^	0.908^**^	0.938^**^	0.851^**^	0.882^**^	0.649^**^

^*^P < 0.05; ^**^P < 0.01^.^ WSOC: soil water-soluble organic carbon; SMBC: soil microbial biomass carbon; SMBN: soil microbial biomass nitrogen; MINC: cumulative CO_2_-C mineralization. See Tables 1 and 2 for abbreviations.

**Table 4:** Linear regression analysis of soil C and N fractions against aboveground, belowground and the total biomass among *P. australis* salt marsh, wheat field and rapeseed field. See Tables 2 and 3 for abbreviations.

	Regression analysis with aboveground biomass			Regression analysis with belowground biomass			Regression analysis with the total biomass		
	Equation	*R* ^2^	*p*	Equation	*R* ^2^	*p*	Equation	*R* ^2^	*p*
SOC (g kg^−1^)	Y = 0.004 X − 1.150	0.633	0.002	Y = 0.001 X + 5.344	0.052	0.478	Y = 0.003 X − 3.436	0.685	0.001
SLOC (g kg^−1^)	Y = 0.002 X − 0.262	0.493	0.011	Y = 0.001 X + 2.317	0.092	0.337	Y = 0.002 X − 1.716	0.646	0.002
SROC (g kg^−1^)	Y = 0.002 X − 0.888	0.707	0.001	Y = 0.001 X + 3.028	0.020	0.658	Y = 0.002 X − 1.720	0.648	0.002
SON (g kg^−1^)	Y = 0.001 X − 0.330	0.683	0.001	Y = 0.001 X + 0.474	0.055	0.464	Y = 0.001 X − 0.610	0.737	0.001
SLON (g kg^−1^)	Y = 0.001 X − 0.330	0.711	0.001	Y = 0.001 X + 0.332	0.042	0.523	Y = 0.001 X − 0.528	0.725	0.001
SRON (g kg^−1^)	Y = 0.001 X + 0.001	0.494	0.011	Y = 0.001 X + 0.142	0.099	0.320	Y = 0.001 X − 0.082	0.659	0.001
WSOC (mg kg^−1^)	Y = 0.017 X + 63.352	0.598	0.003	Y = 0.006 X + 90.524	0.050	0.483	Y = 0.015 X + 53.611	0.651	0.002
SMBC (mg kg^−1^)	Y = 0.066 X + 2.936	0.608	0.003	Y = 0.027 X + 107.421	0.070	0.404	Y = 0.060 X − 41.552	0.708	0.001
SMBN (mg kg^−1^)	Y = 0.019 X + 12.598	0.500	0.010	Y = 0.006 X + 44.729	0.035	0.559	Y = 0.017 X + 2.102	0.526	0.008
MINC (g kg^−1^ 30 day)	Y = 0.140 X + 283.961	0.198	0.147	Y = 0.232 X + 337.784	0.389	0.030	Y = 0.216 X − 67.951	0.666	0.001

## Discussion

Coastal reclamation greatly altered SOC and SON sequestration along Jiangsu coast in eastern China (Table 2; Fig. 2). In this study, the highest levels of SOC, SON, SROC, SLON, and SRON were found in fishpond among land use types (Table 2; Fig. 2). Moreover, SROC, SLON, and SRON were highly related to SOC and SON (Table 3). SOC and SON sequestration is primarily determined by organic residuals entering the soil as well as decomposition of organic matter^29,30^. The fishpond had the highest SOC, SON, SROC, SLON, and SRON among land use types (Table 2; Fig. 2), which was mainly attributed to massive organism excrements and partial residual bodies in fishpond which were decomposed and further adsorbed into the sediment, and greatly promoted SOC, SON, SROC, SLON, and SRON accumulation in fishpond (Table 2; Fig. 2)^29,30^. Generally, high moisture in wetlands contributes to SOM sequestration owing to soil anaerobic environment which is propitious to SOM accumulation for long-term^31^. In the present study, the fishpond was in a long-term flooded state, and showed the highest sediment moisture among land use types (Table 1), that can enhance SOC, SON, SROC, SLON, and SRON sequestration by decreasing decomposition rate of SOM as a result of highly anaerobic conditions in fishpond. This inference was supported by our Pearson’s correlation analyses which indicating that soil moisture was highly related to SOC, SON, SROC, SLON, and SRON (Table 3). Additionally, Chen *et al*.^32^ reported that although most of fish feed they put into the fishpond during their experiments were eaten by fishes, approximately 30% fish feed were not eaten and adsorbed into the sediment after various decomposition processes, and that was considered as one of the reasons for increasing SOC, total N, and total phosphorus in reclaimed aquaculture ponds of the Jiangsu coast. Thus, it is deduced that the highest SOC, SON, SLON, SROC, and SRON in fishpond were mainly caused by large quantities of organic residuals (i.e., organism excrements, partial residual bodies, and fish feed residues) input into the sediment, and lower decomposition of C and N which was associated with the high sediment moisture levels and highly anaerobic conditions in fishpond (Tables 1 and 2; Fig. 2). Additionally, Silveira *et al*.^33^ reported that acid hydrolysis method which was used to determine SROC and SRON through removing carbohydrates and amino acids, and the residues are predominantly left with O-alkyl as well as aromatic C, and these compounds contribute to soil C stabilization^34^. We observed that the recalcitrance indices for soil C (RIC) value (67.7%) was highest in fishpond among land use types (Fig. 2d), suggesting that the SOC of fishpond had the highest proportion of recalcitrant C, and resulting in the most stable SOC pool which was found in fishpond. Meanwhile, the highest recalcitrance indices for soil N (RIN) value (46.93%) was showed in *P. australis* salt marsh, implying that *P. australis* salt marsh had the most stable SON pool among land use types.

Numerous studies have demonstrated that coastal reclamation can change SOC and SON accumulation following coastal wetlands converting into farmlands^8,15,18^. Cui *et al*.^15^ revealed that coastal wetlands which were reclaimed to farmlands greatly decreased SOC and SON sequestration in first 16 years, and then recovered within 30 years, afterward slow accumulated SOC and SON along with cultivation time. Interestingly, in this study, coastal reclamation considerably increased levels of SOC, SON, SLOC, SLON, SROC, and SRON following conversion of *P. australis* salt marsh into wheat and rapeseed fields for 25 years (Table 2; Fig. 2). Our results were supported by Zhang *et al*.^18^ reporting that the concentrations of SOC and soil total N significantly enhanced following conversion of tidal flat into farmlands, such as rice, wheat, and maize fields. Different land use patterns consist of different vegetation types and management practices, which can alter soil water and heat conditions, as well as physiochemical properties^35,36^. Alterations in soil physiochemical properties would greatly affect SOC and SON accumulation^17^. Soil pH and salinity were considered to be the most important factors controlling SOC and SON in coastal reclaimed lands^17,37^. High soil pH decreases solubility of iron, manganese, and zinc that are necessary elements for plant growth^38^, which restricts plant growth and leads to lower amounts of plant materials entering the soil, and ultimately decreases SOC and SON^12^. Soil salinity is one of the crucial drivers of the changes in SOC and SLOC^37,39^. High soil salinity is adverse to SOC and SON accumulation as a result of restriction of plant growth^39^. It has been suggested that high alkalinity and salinity are major features of coastal salt marshes^40,41^, which are also the chief limiting factors for agriculture development in coastal areas^42^. Presently, fresh water irrigation is considered as an effective measure to dealkali and desalt in order to fit the growth of crops following reclamation of coastal wetlands^40,42^. Yin *et al*.^42^ exhibited that salinity of reclaimed soils constantly declined along with continuous freshwater irrigation and ultimately kept a relatively stable level after 60 years of coastal reclamation. This was confirmed by our results suggesting that soil pH and salinity in reclaimed wheat field and rapeseed field significantly decreased compared to those of *P. australis* salt marsh (Table 1). Thus, greatly decreased soil pH and salinity would promote plant growth and increase plant productivity^39^. In this study, aboveground and total biomass in wheat field and rapeseed field significantly increased relative to *P. australis* salt marsh (Fig. 1), and aboveground and total biomass were highly associated with all of SOC and SON fractions (Table 4). Thus, we reasoned that significantly increased crop residuals returning the soil resulted in higher SOC, SON, SLOC, SLON, SROC, and SRON in wheat and rapeseed fields compared to *P. australis* salt marsh (Table 2; Figs 1 and 2)^43^.

In addition to abovementioned nutrient-inputs, organic manure application can directly increase SOC and SON accumulation^43,44^. While chemical fertilizer is able to enhance crop production and ultimately increase crop residuals inputting the soil^44,45^. Consequently, higher levels of SOC and SON and its fractions in wheat and rapeseed fields compared to *P. australis* salt marsh were primarily derived from organic manure application, as well as the abundance of crop residuals input into the soil which was attributed to chemical fertilization as well as lower soil pH and salinity (Tables 1 and 2; Fig. 2). Additionally, SOC, SLOC, SROC, and SRON levels were lowest in town construction land (Table 2; Fig. 2). The loss of these C and N in town construction land have been triggered by the loss of vegetative cover (i.e., *P. australis* community) due to clearing of all the vegetation during coastal reclamation, as well as having no organic or chemical fertilizer applied.

Coastal reclamation greatly changed SOC and SON dynamics along with conversion of *P. australis* salt marsh into different land use types (Fig. 3). The fishpond, wheat and rapeseed fields showed higher WSOC concentration in comparison with *P. australis* salt marsh (Fig. 3). WSOC can be readily metabolized by soil microbes owing to its easily available nutrients and energy^46^. It is widely accepted that WSOC is greatly affected by a wide range of factors, including plant litter, roots, stable organic fractions, and microbial decay products^47^, and types of land use^48,49^. We found that WSOC concentration was highly related to plant aboveground and total biomass, and SOC and SON (Tables 3 and 4). Hence, increased WSOC concentration in wheat and rapeseed fields likely result from input of wheat and/or rapeseed residuals, and exotic organic manure alone or in combination with synthetic nitrogen fertilizers which are attributed to labile organic C inputs (Fig. 3)^49,50^. WSOC concentration was highest in fishpond, primarily owing to the highest SOC and SLOC levels (Table 2, Figs 2 and 3). SMBC and SMBN play significant roles in promoting soil C and N turnover as well as nutrient cycling^51^. SMBC and SMBN concentrations in fishpond, wheat and rapeseed fields were significantly higher than those in *P. australis* salt marsh (Fig. 3b,c). It is inferred that higher WSOC provided more available nutrients and energy for soil microbes (Fig. 3a)^25,46^, which greatly increased soil microbial biomass and led to higher SMBC and SMBN concentrations in fishpond, wheat and rapeseed fields (Fig. 3b,c). Soil C mineralization is greatly affected by adding exogenous materials, e.g., organic residuals^52^. Exogenous materials adding are able to accelerate or restrain soil C mineralization by changing microbial communities’ activities^53^. Our results are consistent with positive priming effects indicating that higher soil/sediment MINC concentration was found in fishpond and wheat field compared with *P. australis* salt marsh (Fig. 3d), was due to higher fresh organic C inputs (Fig. 1), resulting in increased WSOC and higher microbial biomass (i.e., SMBC and SMBN), and thereby increasing SOC decomposition (Fig. 3)^54^. Conversely, WSOC, SMBC, SMBN, and MINC concentrations were lowest in town construction land (Fig. 3), which were the consequence of the reduction of SOC and SON caused by a complete loss of organic residuals inputting into the soil in town construction land after coastal reclamation (Table 2; Fig. 2). Therefore, *P. australis* salt marsh was converted to different land use types that considerably changed SOC and SON dynamics through changing level of WSOC, shifting microbial biomass (i.e., SMBC and SMBN) and modifying C output.

In conclusion, this study emphasized the variations of SOC and SON pools along with coastal reclamation. Our data manifest that coastal reclamation significantly increased stocks/concentrations of SOC, SON, SLOC, SLON, SROC, SRON, as well as WSOC, SMBC, SMBN, and MINC following conversion of *P. australis* salt marsh into fishpond, wheat and rapeseed fields. Whereas, coastal reclamation considerably decreased stocks/concentrations of SOC, SLOC, SROC, SRON, WSOC, SMBC, SMBN, and MINC following conversion of *P. australis* salt marsh into town construction land. Our study investigated the responses of SOC and SON pools and its fractions to short-term coastal reclamation at the ecosystem level. More investigations are needed to assess the long-term impacts of coastal reclamation on ecosystem functioning at region and/or landscape scale, which should consider the spatial stratified heterogeneity at the landscape scale^55,56^. Nevertheless, our results demonstrate that coastal reclamation could greatly affect C and N sinks of coastal wetlands by altering SOC and SON pools size, stability and dynamics changes as a result of the changes in quantity and quality of exogenous materials (e.g., plant materials, organic residuals, organic manure, and chemical fertilizers) returning the soil, and soil physiochemical properties along Jiangsu coast in eastern China. Overall, this study provides new insights to better understand the influence mechanism of coastal reclamation on C and N sinks of coastal wetlands.

## Methods

### Study area

**Figure 4:**
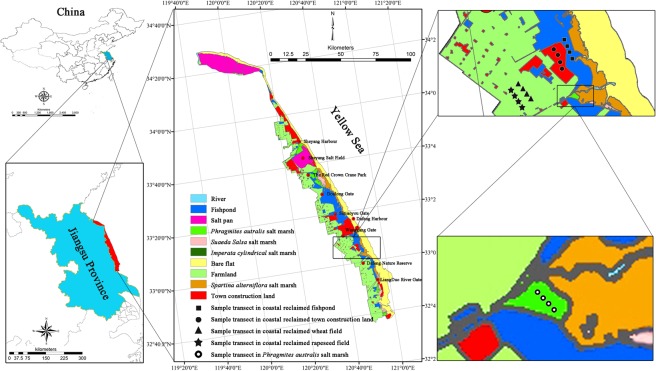
Location of the sampling site in coastal reclaimed fishpond, wheat field, rapeseed field, town construction land, and natural *P. australis* salt marsh along Jiangsu coast in eastern China.

The experiment was conducted in the Yancheng Yellow Sea coast of Jiangsu province, China (Fig. 4). Specific sampling transects were located next to the Dafeng Nature Reserve (32°00′–33°15′ N, 120°40′–121°00′ E) (Fig. 4). The annual mean temperature and precipitation are 14.4 °C and 1088 mm, respectively^28^. The natural vegetations in Yancheng Yellow Sea coast are listed from sea to inland: *S. alterniflora*, *S. salsa*, *Imperata cylindrica*, and *P. australis* salt marshes (Fig. 4)^24^. In the past century, the coastal wetlands of Jiangsu coast have experienced intensive reclamation^12^. Currently, the majority of coastal wetlands have been reclaimed and converted into the fishponds, farmlands, and town construction lands, especially in Dafeng and Sheyang counties (Fig. 4)^26,28^. Wheat (*Triticum aestivum* L.) field, and rapeseed (*Brassica campestris* L.) field are the most common and widely distributed farmlands along the middle Jiangsu coast. *P. australis* salt marshes are the easiest to be reclaimed into other land use types due to growing further inland and the farthest from the sea compared to *S. alterniflora*, *S. salsa*, and *I. cylindrica* salt marshes (Fig. 4)^22^.

### Sample collection

In June 2016, four sample transects of 40 m × 40 m were selected in each land use type, i.e., *P. australis* salt marsh (the control), fishpond, wheat field, rapeseed field, and town construction land (Fig. 4), respectively, and there was a distance of 100 m apart between the two adjacent sample transects in each land use type. The historical records and Landsat Thematic Mapper satellite images (1975, 1991, 2000, 2006, 2010, and 2013 year) of Yancheng Yellow Sea coast of Jiangsu province were analyzed to identify the reclamation time of land use types and the types of natural salt marsh before coastal reclamation in the sampling region. The fishpond, wheat field, and rapeseed field in the sample transects have been reclaimed for approximately 25 years, they were *P. australis* salt marshes before coastal reclamation. The town construction land of the sample transects has been established for 6 years, which suffered continual coastal reclamation from *P. australis* salt marsh in 1975 to fishpond in 1991, and further converted into town construction land in 2010. Due to large area of *P. australis* salt marshes have been reclaimed to farmlands, fishponds, and town construction lands, and only a small area of *P. australis* salt marsh remained in the sampling region (Fig. 4). In this study, we randomly selected three 2 m × 2 m plots in each transect, and three points were chose for the collection of soil samples from each plot. Subsequently, soil samples from each plot were thoroughly mixed to yield a final soil sample. We randomly established three 0.5 m × 0.5 m quadrats to gather aboveground plant materials, and dug three soil blocks (0.15 m long × 0.15 m wide × 0.30 m deep) to gather root materials in each transect of *P. australis* salt marsh, and wheat and rapeseed fields.

### Laboratory analysis

Specific methods of collecting belowground biomass (i.e., roots) from soil blocks were described by Yang *et al*.^25^. All plant samples were cleaned and dried using oven at 65 °C for 48 h to constant weight to examine aboveground, belowground, as well as total biomass. Soil bulk density was examined through the method of cutting ring^25^. The determination of soil moisture was based on the method of our previously described^24^. Plant and animal debris in soil samples was eliminated, and soil samples were sufficiently mixed and divided into two subsamples. The first soil subsample was air-dried and sifted using a 1 mm sieve for determination of soil pH, salinity, SOC, SROC, SON, and SRON. The second soil subsample was sifted using a 0.25 mm sieve and preserved at 4 °C for measurement of WSOC, SMBC, SMBN, and MINC. Soil pH was analyzed in a 1:2.5 soil/water suspension using a digital pH meter^25^. Soil salinity was determined in a 1:5 soil/water suspension^24^.

Soil recalcitrant SOM pool was determined by acid hydrolysis method described by Rovira and Vallejo (2000)^21^ and Yang *et al*.^22^. Oven-dried soil subsamples were treated with 1 M HCl for 24 h at room temperature to eliminate all inorganic C and N, and the unhydrolyzed fraction was considered as the SOM pool^22^. The 1 g of SOM sample was placed into a 100 mL round-bottom flask and was hydrolyzed with 25 mL of 6 M HCl at 105 °C for 18 h in sealed Pyrex tubes. After cooling, unhydrolyzed residues were gathered through centrifugation and decantation of the supernatant using deionized water to get rid of remanent HCl, the residues were oven-dried at 60 °C to a constant weight and the unhydrolyzed residues considered as recalcitrant SOM pool^21^. The concentrations of C and N in the SOM pool (SOC and SON, respectively) and recalcitrant SOM pool (SROC and SRON, respectively) were examined by an Elementar Vario Micro CHNS analyzer (Elementar Analysensystem GmbH, Germany). The recalcitrance indices for soil C and N (RIC and RIN, respectively) were calculated by the following equations^21^: RIC (%) = (unhydrolyzed C/total OC) × 100; RIN (%) = (unhydrolyzed N/total ON) × 100.

WSOC was determined in accordance with our previously described methods^24^. SMBC and SMBN were measured by chloroform fumigation extraction method^57^. MINC was analyzed by a laboratory soil aerobic incubation experiment^58,59^. Briefly, fresh soil samples (equivalent to 20 g dry weight) were placed in 50 mL glass beaker. Ultrapure water was added to soil samples to maintain moisture at 60% of their water-holding capacity. The glass beaker was placed in a 500 mL wild-mouth bottle, and polyethylene tubes including10 mL of a 0.5 M NaOH solution was placed in each wild-mouth bottle to absorb CO_2_ evolved by the soil. The wild-mouth bottles were sealed and incubated at 28 °C in the dark for 30 days. After incubation for 5, 10, 15, 20, 25, and 30 days, took out polyethylene tubes containing NaOH. Then the wild-mouth bottles were opened for a few minutes to keep up sufficient O_2_ levels. The CO_2_ emitted was measured by titration of the NaOH solution with 0.1 M HCl in two drops BaCl_2_.

### Statistical analysis

The concentrations and stocks of C and N in labile SOM pool (SLOC and SLON, respectively) were obtained by subtracting C and N of recalcitrant SOM pool from SOM pool in statistically paired samples. One-way ANOVAs was applied to evaluate the impacts of coastal reclamation on soil moisture, bulk density, pH, salinity, SOC, SLOC, SROC, SON, SLON, SRON, WSOC, SMBC, SMBN, and MINC; RIC and RIN ratios; and the aboveground, belowground, and total biomass with the SPSS 22 statistical software. Pearson’s correlation analysis was performed to correlate various SOC and SON fractions with the soil and plant properties. Linear regression analysis was performed to correlate various soil C and N fractions with aboveground, belowground, and total biomass among *P. australis* salt marsh, wheat and rapeseed fields.

## References

[1] Feng L (2015). Evaluation for coastal reclamation feasibility using a comprehensive hydrodynamic framework: A case study in Haizhou Bay. Mar. Pollut. Bull..

[2] Hoeksema RJ (2007). Three stages in the history of land reclamation in The Netherlands. Irrig. Drain..

[3] Lin QY, Yu S (2018). Losses of natural coastal wetlands by land conversion and ecological degradation in the urbanizing Chinese coast. Sci. Rep..

[4] Wang W, Liu H, Li YQ, Su JL (2014). Development and management of land reclamation in China. Ocean Coast. Manage..

[5] Cramer W (2017). Biodiversity and food security: from trade-offs to synergies. Reg. Environ. Change.

[6] Chen WG (2017). Monitoring and analysis of coastal reclamation from 1995–2015 in Tianjin Binhai New Area, China. Sci. Rep..

[7] Bernal B, Mitsch WJ (2012). Comparing carbon sequestration in temperate freshwater wetland communities. Global Change Biol..

[8] Crooks, S., Herr, D., Tamelander, J., Laffoley, D. & Vandever, J. Mitigating climate change through restoration and management of coastal wetlands and near-shore marine ecosystems: challenges and opportunities. World Bank, Washington DC, Environment Department Paper **121** (2011).

[9] Bu NS (2015). Reclamation of coastal salt marshes promoted carbon loss from previously-sequestered soil carbon pool. Ecol. Eng..

[10] Cheong S (2013). Coastal adaptation with ecological engineering. Nat. Clim. Change.

[11] Saunders MJ, Kansiime F, Jones MB (2012). Agricultural encroachment: implications for carbon sequestration in tropical African wetlands. Global Change Biol..

[12] Zhang H (2018). Dynamics and driving factors of the organic carbon fractions in agricultural land reclaimed from coastal wetlands in eastern China. Ecol. Indic..

[13] Li J (2014). Evolution of soil properties following reclamation in coastal areas: a review. Geoderma.

[14] Setia R (2012). Simulation of salinity effects on past, present, and future soil organic carbon stocks. Environ. Sci. Technol..

[15] Cui J (2012). Long-term changes in topsoil chemical properties under centuries of cultivation after reclamation of coastal wetlands in the Yangtze Estuary, China. Soil Till. Res..

[16] Chuai XW, Huang XJ, Wang WJ, Wu CY, Zhao RQ (2014). Spatial simulation of land use based on terrestrial ecosystem carbon storage in coastal Jiangsu, China. Sci. Rep..

[17] Deng X (2016). Soil organic carbon of an intensively reclaimed region in China: current status and carbon sequestration potential. Sci. Total Environ..

[18] Zhang H (2016). Organic carbon and total nitrogen dynamics of reclaimed soils following intensive agricultural use in eastern China. Agr. Ecosyst. Environ..

[19] Han GX (2014). Agricultural reclamation effects on ecosystem CO2 exchange of a coastal wetland in the Yellow River Delta. Agr. Ecosyst. Environ..

[20] Novara, A. *et al*. Turnover and availability of soil organic carbon under different Mediterranean land-uses as estimated by ^13^C natural abundance. *Eur. J. Soil Sci.* **64**, 466–475 (2013).

[21] Rovira P, Vallego VR (2000). Examination of thermal and acid hydrolysis procedures in characterization of soil organic matter. Commun. Soil Sci. Plan..

[22] Yang W (2015). Labile and recalcitrant soil carbon and nitrogen pools in tidal salt marshes of the eastern Chinese coast as affected by short-term C4 plant *Spartina alterniflora* invasion. Clean–Soil, Air, Water.

[23] McLauchlan KK, Hobbie SE (2004). Comparison of labile soil organic matter fractionation techniques. Soil Sci. Soc. Am. J..

[24] Yang W (2013). Consequences of short-term C4 plant *Spartina alterniflora* invasions for soil organic carbon dynamics in a coastal wetland of Eastern China. Ecol. Eng..

[25] Yang W (2016). The impact of sea embankment reclamation on soil organic carbon and nitrogen pools in invasive *Spartina alterniflora* and native *Suaeda salsa* salt marshes in eastern China. Ecol. Eng..

[26] Long XH, Liu LP, Shao TY, Shao HB, Liu ZP (2016). Developing and sustainably utilize the coastal mudflat areas in China. Sci. Total Environ..

[27] Meng WQ (2017). Temporal-spatial variations and driving factors analysis of coastal reclamation in China. Estuar. Coast. Shelf S..

[28] Cai FF, Vliet JV, Verburg PH, Pu LJ (2017). Land use change and farmer behavior in reclaimed land in the middle Jiangsu coast, China. Ocean Coast. Manage..

[29] Poeplau C, Don A (2013). Sensitivity of soil organic carbon stocks and fractions to different land-use changes across Europe. Geoderma.

[30] Liu X, Li LH, Qi ZM, Han JG, Zhu YL (2017). Land-use impacts on profile distribution of labile and recalcitrant carbon in the Ili River Valley, northwest China. Sci. Total Environ..

[31] Whitting GJ, Chanton JP (2001). Greenhouse carbon balance of wetlands: methane emission versus carbon sequestration. Tellus B.

[32] Chen GX, Gao DZ, Wang ZP, Zeng CS (2017). Contents of carbon, nitrogen and phosphorus in sediments in aquaculture ponds for different reclamation years in Shanyutan wetlands and its pollution risk assessment. Wetland Sci..

[33] Silveira ML, Comerford NB, Reddy KR, Cooper WT, El-Rifai H (2008). Characterization of soil organic carbon pools by acid hydrolysis. Geoderma.

[34] Pandey D, Agrawal M, Bohra JS, Adhya TK, Bhattacharyya P (2014). Recalcitrant and labile carbon pools in a sub-humid tropical soil under different tillage combinations: A case study of rice–wheat system. Soil Till. Res..

[35] Wallenius K (2011). Effects of land use on the level, variation and spatial structure of soil enzyme activities and bacterial communities. Soil Biol. Biochem..

[36] Xie XF (2017). Response of soil physicochemical properties and enzyme activities to long-term reclamation of coastal saline soil, Eastern China. Sci. Total Environ..

[37] Rath KM, Rousk J (2015). Salt effects on the soil microbial decomposer community and their role in organic carbon cycling: a review. Soil Biol. Biochem..

[38] Grybos M, Davranche M, Gruau G, Petitjean P, Pédrot M (2009). Increasing pH drives organic matter solubilization from wetland soils under reducing conditions. Geoderma.

[39] Setia R (2013). Soil salinity decreases global soil organic carbon stocks. Sci. Total Environ..

[40] Iost S, Landgraf D, Makeschin F (2007). Chemical soil properties of reclaimed marsh soil from Zhejiang Province P.R. China. Geoderma.

[41] Li X, Sun Y, Mander Ü, He Y (2013). Effects of land use intensity on soil nutrient distribution after reclamation in an estuary landscape. Landscape Ecol..

[42] Yin A (2016). Salinity evolution of coastal soils following reclamation and intensive usage, Eastern China. Environ. Earth Sci..

[43] Chatterjee S, Bandyopadhyay KK, Pradhan S, Singh R, Datta SP (2018). Effects of irrigation, crop residue mulch and nitrogen management in maize (*Zea mays* L.) on soil carbon pools in a sandy loam soil of Indo-gangetic plain region. Catena.

[44] Steiner C (2007). Long term effects of manure, charcoal andmineral fertilization on crop production and fertility on a highly weathered Central Amazonian upland soil. Plant Soil.

[45] Kalbitz K (2013). The carbon count of 2000 years of rice cultivation. Global Change Biol..

[46] Rodríguez-Caballero, G. *et al*. Striking alterations in the soil bacterial community structure and functioning of the biological N cycle induced by *Pennisetum setaceum* invasion in a semiarid environment. *Soil Biol. Biochem.* **109**, 176–187 (2017).

[47] Sanderman J, Baldock JA, Amundson R (2008). Dissolved organic carbon chemistry and dynamics in contrasting forest and grassland soils. Biogeochemistry.

[48] Chantigny MH (2003). Dissolved and water-extractable organic matter in soils: a review on the influence of land use and management practices. Geoderma.

[49] Li SQ (2018). Influences of observation method, season, soil depth, land use and management practice on soil dissolvable organic carbon concentrations: A meta-analysis. Sci. Total Environ..

[50] Marschner B, Kalbitz K (2003). Controls of bioavailability and biodegradability of dissolve organic matter in soils. Geoderma.

[51] Zuber SM, Villamil MB (2016). Meta-analysis approach to assess effect of tillage on microbial biomass and enzyme activities. Soil Biol. Biochem..

[52] Hamer U, Marschner B (2002). Priming effects of sugars, amino acids, organic acids and catechol on the mineralization of lignin and peat. J. Plant Nutr. Soil Sc..

[53] Sullivan BW, Hart SC (2013). Evaluation of mechanisms controlling the priming of soil carbon along a substrate age gradient. Soil Biol. Biochem..

[54] Xiao D (2018). Soil organic carbon mineralization with fresh organic substrate and inorganic carbon additions in a red soil is controlled by fungal diversity along a pH gradient. Geoderma.

[55] Wang JF, Zhang TL, Fu BJ (2016). A measure of spatial stratified heterogeneity. Ecol. Indic..

[56] Wang JF, Xu CD (2017). Geodetector: Principle and prospective. Acta Geogr. Sin..

[57] Vance ED, Brookes PC, Jenkinson DS (1987). An extraction method for measuring microbial biomass C. Soil Biol. Biochem..

[58] Zhang W (2005). Soil microbial responses to experimental warming and clipping in a tallgrass prairie. Global Change Biol..

[59] Nie M (2013). Positive climate feedbacks of soil microbial communities in a semi-arid grassland. Ecol. Lett..

